# Temporary sphincter-preserving covered biliary stent: a novel adjunctive device for cholangioscopy-guided calculi clearance

**DOI:** 10.1055/a-2674-4861

**Published:** 2025-09-04

**Authors:** Wengang Zhang, Hongyi Sun, Qingzhen Wu, Zhenyu Liu, Bozong Shao, Enqiang Linghu

**Affiliations:** 1 104607 Department of Gastroenterology, The First Medical Center of Chinese PLA General Hospital, Beijing, China

To date, cholangioscopy-guided calculi clearance has become a well-established treatment method for common bile duct (CBD) calculi 1 2 . However, these procedures typically require endoscopic sphincterotomy (EST), which might lead to the loss of sphincter of Oddi (SO) function and some adverse events, including bleeding, perforation, cholangitis, and calculi recurrence 3 . Importantly, EST was not appropriate for those patients who could not stop taking anticoagulation/antiplatelet agents. Therefore, multiple investigators – including our team – have pioneered the deployment of a self-expandable metal stent (SEMS) prior to calculi clearance as an alternative sphincter-preserving strategy 4 5 . However, clinical experience reveals that calculi frequently become lodged between the SEMS and biliary wall ( Fig. 1 ), presenting a critical limitation to the widespread adoption of this strategy.

**Fig. 1:**
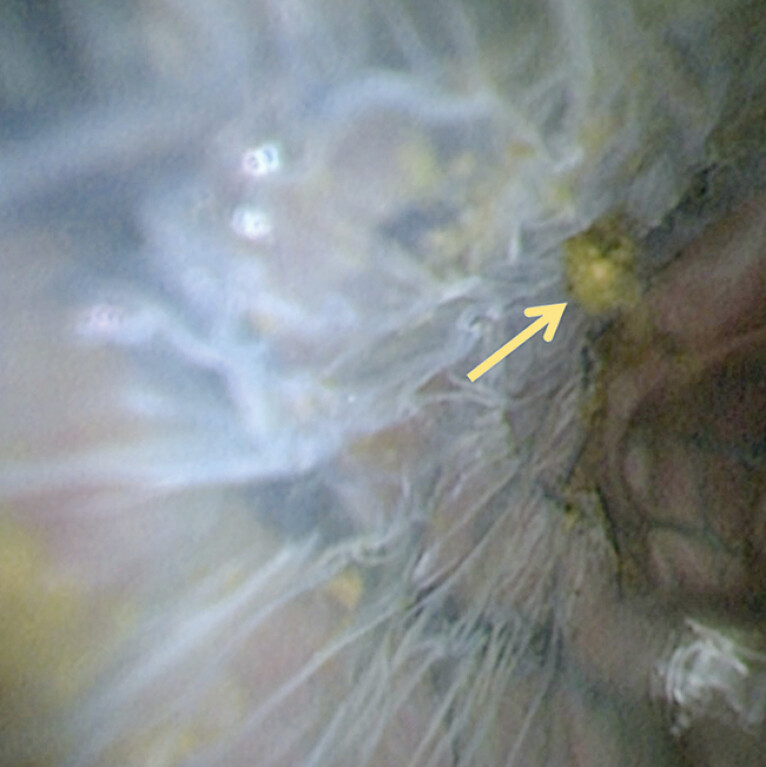
Calculi entrapment between the self-expandable metal stent (SEMS) and the biliary wall.

In this study, we introduce an innovative temporary sphincter-preserving covered biliary stent (TSP-CBS) ( Fig. 2 ) in a porcine model, engineered to prevent calculi entrapment between the SEMS and biliary wall, enabling EST-free cholangioscopy-guided calculi clearance. First, biliary intubation was conducted in a porcine model. Second, the TSP-CBS was advanced over the guidewire to the distal CBD, followed by deployment of its distal umbrella-shaped occlusive device at a predetermined position distal to the target calculi ( Fig. 3 ). Third, the TSP-CBS was advanced to its predetermined deployment site, during which the target calculi was proximally displaced into upstream CBD by the expanded umbrella-shaped occlusive device ( Fig. 4 ). Fourth, the TSP-CBS was deployed while eliminating calculi entrapment risk between the stent and biliary wall. Fifth, the umbrella-shaped occlusive device was collapsed and retrieved. Sixth, cholangioscope cannulation was performed through the TSP-CBS ( Fig. 5 ), revealing intact biliary mucosa without iatrogenic trauma. Finally, the deployed TSP-CBS was extracted using a snare ( Video 1 ).

**Fig. 2:**
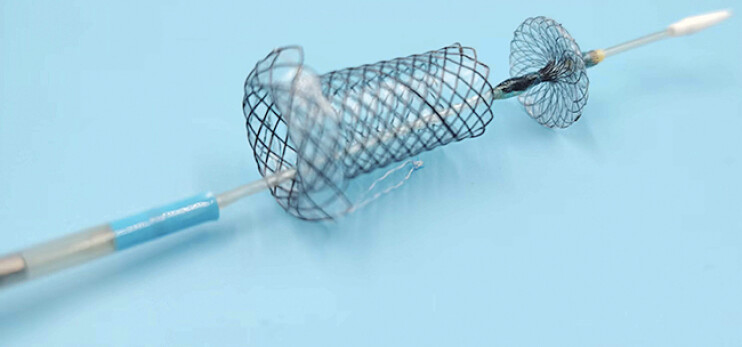
The innovative temporary sphincter-preserving covered biliary stent (TSP-CBS).

**Fig. 3:**
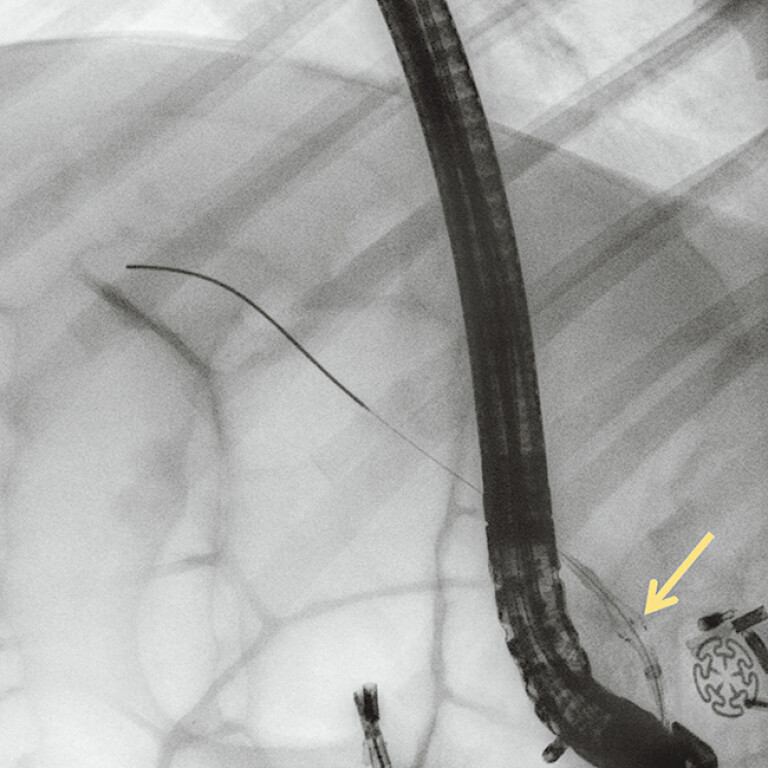
The temporary sphincter-preserving covered biliary stent (TSP-CBS) was advanced over the guidewire to the distal common bile duct (CBD), followed by deployment of its distal umbrella-shaped occlusive device at a predetermined position distal to the target calculi.

**Fig. 4:**
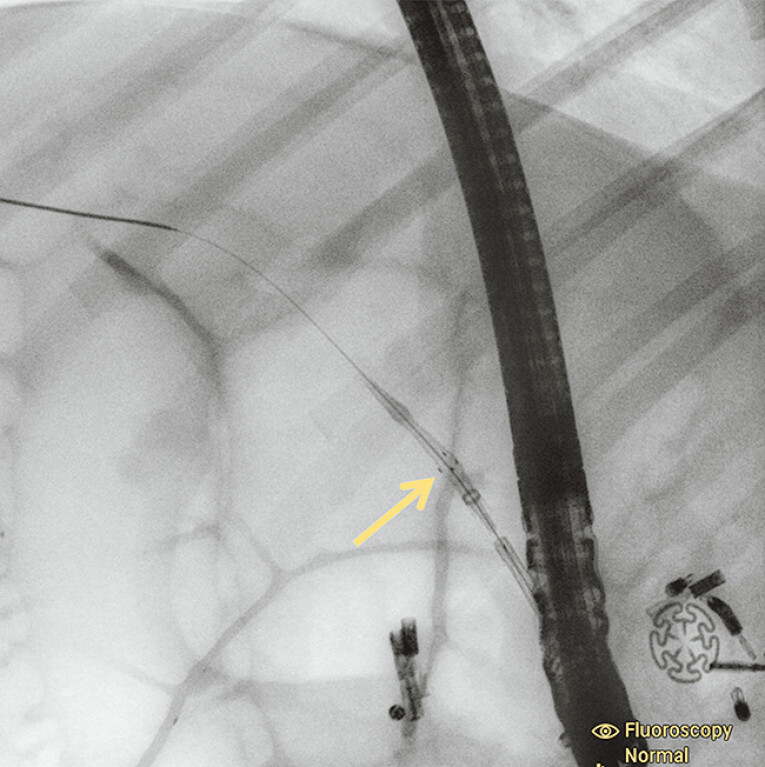
The temporary sphincter-preserving covered biliary stent (TSP-CBS) was advanced to its predetermined deployment site, during which the target calculi were proximally displaced into the upstream common bile duct (CBD) by the expanded umbrella-shaped occlusive device.

**Fig. 5:**
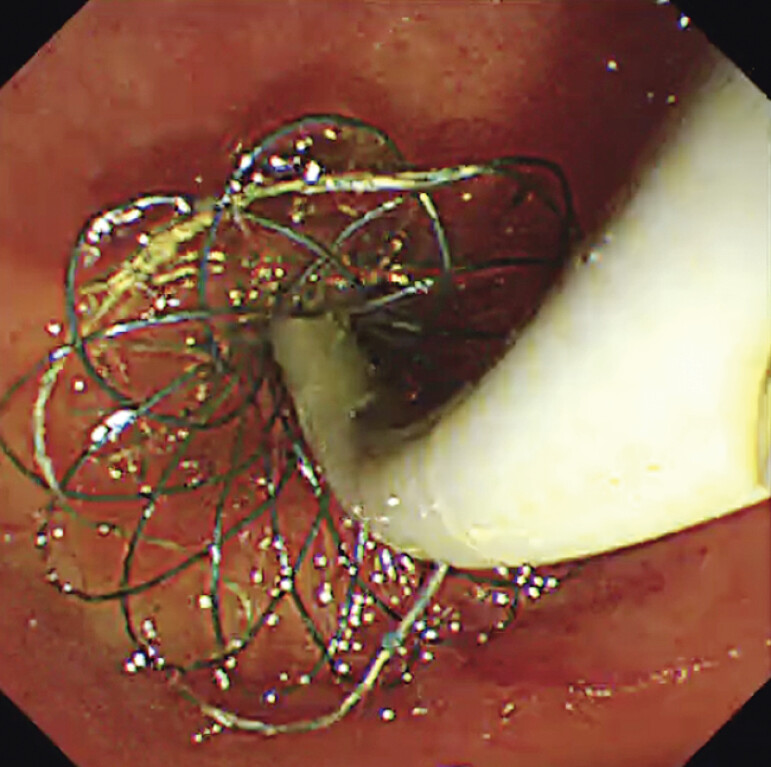
Cholangioscope cannulation was performed through the temporary sphincter-preserving covered biliary stent (TSP-CBS).

The procedure of endoscopic sphincterotomy (EST)-free cholangioscopy-guided calculi clearance using the temporary sphincter-preserving covered biliary stent (TSP-CBS) in a porcine model.Video 1

This preclinical study validated the feasibility of EST-free cholangioscopy-guided calculi clearance using the TSP-CBS. Subsequent clinical validation will be pursued to translate this sphincter-preserving strategy.

Endoscopy_UCTN_Code_TTT_1AR_2AH

## Notes

Endoscopy E-Videos https://eref.thieme.de/e-videos
*E-Videos* is an open access online section of the journal *Endoscopy* , reporting on interesting cases and new techniques in gastroenterological endoscopy. All papers include a high-quality video and are published with a Creative Commons CC-BY license. Endoscopy E-Videos qualify for HINARI discounts and waivers and eligibility is automatically checked during the submission process. We grant 100% waivers to articles whose corresponding authors are based in Group A countries and 50% waivers to those who are based in Group B countries as classified by Research4Life (see: https://www.research4life.org/access/eligibility/ ). This section has its own submission website at https://mc.manuscriptcentral.com/e-videos .
